# The inclusion of a partial meal replacement with or without inulin to a calorie restricted diet contributes to reach recommended intakes of micronutrients and decrease plasma triglycerides: A randomized clinical trial in obese Mexican women.

**DOI:** 10.1186/1475-2891-11-44

**Published:** 2012-06-18

**Authors:** Alma Rosa Tovar, María del Carmen Caamaño, Sandra Garcia-Padilla, Olga Patricia García, Miguel Angel Duarte, Jorge L Rosado

**Affiliations:** 1 School of Natural Sciences, University of Queretaro, Queretaro, Mexico; 2 Cindetec A.C, Queretaro, Mexico; 3 Facultad de Ciencias Naturales, Universidad Autónoma de Querétaro, Av Ciencias S/N Juriquilla, Querétaro, Qro, 76230, Mexico

**Keywords:** Obesity, Inulin, Partial meal replacement, Lipids

## Abstract

**Background:**

Obesity is a major public health problem in many poor countries where micronutrient deficiencies are prevalent. A partial meal replacement may be an effective strategy to decrease obesity and increase micronutrient intake in such populations. The objective was to evaluate the efficacy of a partial meal replacement with and without inulin on weight reduction, blood lipids and micronutrients intake in obese Mexican women.

**Methods:**

In a randomized controlled clinical trial 144 women (18–50 y) with BMI ≥ 25 kg/m^2^, were allocated into one of the following treatments during 3 months: 1) Two doses/d of a partial meal replacement (PMR), 2) Two doses/d of PMR with inulin (PMR + I) , 3) Two doses/d of 5 g of inulin (INU) and 4) Control group (CON). All groups received a low calorie diet (LCD). Weight, height, hip and waist circumference were measured every 2 weeks and body composition, lipids and glucose concentration and nutrient intake were assessed at baseline and after 3 months.

**Results:**

All groups significantly reduced weight, BMI, waist and hip circumference. Differences between groups were only observed in BMI and weight adjusted changes: At 45 days PMR group lost more weight than INU and CON groups by 0.9 and 1.2Kg, respectively. At 60 days, PMR + I and PMR groups lost more weight than in INU by 0.7 and 1Kg, respectively. Subjects in PMR, PMR + I and INU significantly decreased triglycerides. Energy intake was reduced in all groups. Fiber intake increased in PMR + I and INU groups. Some minerals and vitamins intakes were higher in PMR and PMR + I compared with INU and CON groups.

**Conclusion:**

Inclusion of PMR with and without inulin to a LCD had no additional effect on weight reduction than a LCD alone but reduced triglycerides and improved intake of micronutrients during caloric restriction. PMR could be a good alternative for obese populations with micronutrient deficiencies.

**ClinicalTrials.Gov ID:**

NCT01505023

## Introduction

The prevalence of obesity is increasing in many countries and has become a public health challenge in populations worldwide. It is estimated that at least one billion of individuals worldwide are overweight (body mass index, BMI ≥ 25 kg/m^2^) and at least 300 million are obese (BMI ≥ 30 kg/m^2^) [1,2]. The prevalence of obesity has specially increased in populations in developing countries; for example, a recent survey carried out in Mexico found that the prevalence of obesity in adults in 2006 was 24.2% in men and 34.5% in women, increasing 4% in only 6 years [3,4].

Recent studies have suggested that micronutrient deficiencies might be associated with a higher susceptibility for obesity which might partially explain why obesity in poor countries has increased more rapidly [5]. The possible association of some micronutrient deficiencies with a higher susceptibility for fat deposition and obesity has recently been revised [6]. Since calorie restricted diets might worsen micronutrient deficiencies, a strategy to reduce calories in obese population with a high probability of having some micronutrient deficiencies, should contemplate increasing the intake of vitamins and minerals.

Low calorie diets (LCD) and an increase in physical activity are effective strategies to reduce body fat and to control body weight [7]; however a high proportion of individuals trying to lose weight find it very difficult to reduce energy intake [8]. Partial meal replacements (PMR) are single food or selection of foods intended as replacement of one or two daily meals to reduce energy intake. PMR are made with variety of food preparations, they can be liquid or powder formulas, frozen foods or bars fortified, all fortified with vitamins and minerals [9]. In addition to energy intake reduction, PMR have been used to increase the intake of vitamins, minerals and proteins and thus decrease the risk of deficiencies that are common during energy restriction diets [10,11]. Several studies have evaluated the effectiveness of PMR to reduce weight among obese individuals with different results [10,12]. Most of these studies however, have been carried out in developed populations with a low risk of having micronutrients deficiencies.

Besides the addition of vitamins and minerals, the effectiveness of PMR may be improved by being used as a vehicle for substances with a potential benefit for the obese. Inulin is a non-digestible and non-flavored polisaccharide with low energy content that has been shown to have a positive effect on lipids metabolism [13-20]. A recent meta-analysis by Brighenti [21] reviewed 15 studies and concluded that supplementing individuals with 7 to 10 g/d of inulin reduces their serum triglycerides by 7.5%. A reduction of triglycerides in obese patients would be beneficial since about 36% of individuals with obesity have high serum triglycerides [22]. It has also been suggested that inulin may increase the absorption of minerals such as zinc, iron, magnesium and calcium [23].

The objective of this study was to test the efficacy of a PMR added with vitamins, minerals and inulin on weight reduction, blood lipids and micronutrients intake in obese Mexican women.

## Methods

### Subjects and treatments

A total of 144 non-pregnant, non-lactating, overweight or obese women (BMI ≥ 25 kg/m ^2^) were included in the study. The sample size considered an alpha error of 0.05, statistical power of 0.80 and a lost to follow up rate of 20% to detect a difference in body weight reduction of 4% between treatments and control with an estimated standard deviation of 6%. Women were excluded if they had been previously diagnosed with diabetes, hypertension or if they had fasting glucose ≥126 mg/dL and blood triglycerides ≥400 mg/dL. All subjects received oral and written information of the study protocol, including benefits and potential risks, and voluntarily accepted to participate in the study. The study protocol was reviewed and approved by the Bioethics Committee of the University of Queretaro.

The study was a randomized, controlled, longitudinal, clinical trial. Women who accepted to participate were randomized into one of 4 groups of 36 women each, with a computer generated randomization list. Women in each group received during 3 months one of the following treatments: 1) PMR alone; 2) PMR + 10 g of inulin (PMR + I); 3) 10 g of inulin alone (INU) or 4) Control group with no additional treatment (CON). In addition, a LCD was provided to subjects in all 4 groups. The LCD consisted of educating subjects in detail with a printed guideline developed according with the official food guide for Mexican population (NOM-043-SSA2-2005) and with the National Institutes of Health guide to treat overweight and obese adults [24]. The guide showed the appropriate proportions of the different food groups in a meal and included a list of foods with high content of fat and/or sugar that should be avoided and replaced by equivalent low calorie foods (i.e. whole milk for skimmed milk). The guide also provided suggestions to include high fiber products and water and the size of food portions to include in the diet.

The PMR was developed before the initiation of the clinical trial by a pharmaceutical laboratory located in the city of Queretaro where the study was carried out ( Esbelia^TM^). The PMR was designed to contain sufficient amounts of all vitamins and minerals, especially those that have been reported to be deficient in Mexicans [25]. The nutrient composition of the PMR used in this study is shown in Table 1. An additional version of the PMR was also developed which contained 5 g of inulin per dose; inulin was obtained from Beneo^TM^ Orafti. Treatments PMR and PMR + I were delivered to women in can containers with 400 grams of products every other week and were available in chocolate and vanilla flavors. Women were instructed to consume 2 servings per day of the respective treatment at breakfast and dinner, each consisting of 33 g of powder dissolved in 250 ml of skim milk. Subjects were instructed not to consume any additional foods for breakfast or dinner. Women in the INU group received two can containers with 100 g of inulin every two weeks; they were asked to consume one 5 g serving of inulin mixed with any drink at breakfast and same amount at dinner (10 g of inulin/d).

**Table 1:** Nutrient composition of one dose of a partial meal replacement (PMR) as powder and prepared with skim milk

**Nutrients**	**PMR powder**	**PMR Prepared with skim milk**
	33 g	273 g
**MACRONUTRIENTS**		
Energy, Kj	483.79	835.25
Proteins, g	5.38	13.54
Carbohydrates, g	17.49	29.25
Lipids, g	0.40	0.78
Dietary fiber, g	0.12	0.12
**MICRONUTRIENTS**		
**Vitamins**		
A (Retinol), μg	199.50	345.90
D (Cholecalciferol), μg	2.50	2.50
E, mg	9.10	9.10
K, μg	26.25	26.25
C, mg	52.50	54.42
Folic Acid, μg	230.00	242.72
B_1_, mg	0.45	0.54
B_2_, mg	0.45	0.78
B_6_, mg	0.50	0.60
B_12_, μg	1.20	2.11
Niacin, mg	6.00	6.19
Biotin, μg	15.00	15.00
Pantotenic acid, mg	2.50	2.50
**Minerals**		
Calcium, mg	180.00	475.20
Phosphorus, mg	92.74	92.74
Iodine, μg	25.00	25.00
Iron, mg	5.10	5.19
Magnesium, mg	75.00	101.40
Cupper, mg	0.22	0.22
Zinc, mg	3.30	4.26
Manganese, mg	0.54	0.54
Potassium, mg	7.70	406.10
Sodium, mg	20.69	143.09
Selenium, μg	14.40	14.40
Chromium, μg	6.60	6.60
Molybdenum, μg	13.50	13.50
PMR = Partial meal replacement		

The participants were followed by visits to the clinic every two weeks and by a phone interview once every week. During the first visit, women in the PMR, PMR + I and INU groups received oral and written information explaining how to prepare the treatments and how to follow the LCD. Women in the control group only received the LCD. At every visit, women were asked to bring empty cans or cans with remaining product to exchange for new ones. During the visits at the clinics and during telephone calls, nutritionists reviewed the LCD guide again with the subjects and answered any questions they might have.

Women in PMR, PMR + I and INU treatment groups filled an adherence log dairy to record the consumption of treatments; they were asked to register if they consumed or did not consume the treatment at breakfast and dinner. Adherence to treatments was defined as the number of days that each treatment was consumed divided by the total number of days that each subject was followed.

### Anthropometry and body composition

Weight, height, hip and waist circumference were measured at baseline and every 2 weeks during three months. Measurements were carried out by trained nutritionists following standard procedures [26]. Weight was measured to the nearest 100 g using electronic scales (SECA ONDA 843, Hamburg, Germany). Height was measured with a stadiometer (SECA 206, Hanover, MD) to the nearest 0.1 cm. Waist and hip circumference was measured to the nearest 0.1 cm with a flexible band (SECA 200; Seca Deutschland) following procedures recommended by WHO [27]. Measurements on each subject on different occasions were done by the same examiner to reduce variability.

Body composition was determined with bioelectrical impedance (Quantum X BIA, RJL Systems Inc. Clinton Township, MI) at baseline and every month during the three months of follow-up. Women were asked to lay down in a quiet room and remain calmed for the duration of the measurement. Fat percent was calculated from resistance and reactance using Kotler equations [28].

### Glucose and lipid profile

A fasting blood sample was taken at baseline and after 3 months to measure plasma lipids and glucose concentrations. Plasma glucose was determined using the glucose oxidase method. Total serum cholesterol, HDL-cholesterol, and triglycerides were measured using a colorimetric method with an auto analyzer RA 50 (Johnson&Johnson Vitros DT60 II Chemistry System Rochester, NY USA). LDL was estimated with the Friedewald equations [29].

### Food and nutrient intake evaluation

Food intake was assessed at baseline and after three months using three 24-recalls, one was applied on a weekend day and two on days of the week, Nutrient intake was determined using food composition tables from the US department of agriculture [30] and from the National Institute of Medical Sciences and Nutrition [31]. Percents of adequacy were calculated from the recommended nutrients intake for Mexican population [32,33].

### Statistical analysis

Descriptive analysis was carried out for all variables. Within treatment change in anthropometry, body fat and lipids concentration, by treatment group, were compared with paired T-test; this test was used because the independent variable has two levels (pre-treatment and post-treatment). To evaluate the effect of treatment on anthropometry and lipids concentration an analysis of variance (ANOVA) adjusted for initial values was carried out considering the change (final-initial value) as the dependent variable. Unadjusted ANOVA to evaluate nutrients intake was used to compare between treatments. All reported p-values are based on two-sided tests and compared to a significance level of 0.05. SPSS version 10 (SPSS Inc, Chicago, IL, U.S.A.) software was used for all statistical calculations.

## Results

**Figure 1:**
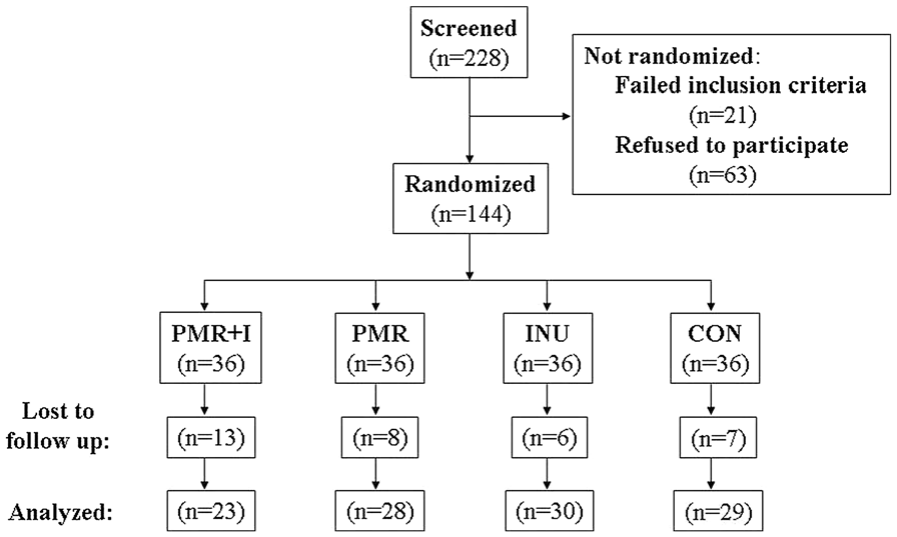
Flow-chart.

Flow diagram of participants is shown in Figure 1. The subjects that did not finish the intervention, abandoned the study for personal reasons not related to the treatment. Characteristics of subjects by treatment group at the beginning of the study are described in Table 2. No differences were observed between groups in any of the study variables at baseline or between subjects that were lost to follow up and subjects that remained to the end of the study.

**Table 2:** **Characteristics of subjects at baseline by treatment group**^**1,2**^

	**PMR + I**	**PMR**	**INU**	**CON**
N	23	28	30	29
Age, y	34.62 ± 7.4	33.17 ± 7.63	32.58 ± 8.13	33.39 ± 8.72
Weight, Kg	75.96 ± 11.71	75.79 ± 12.45	76.55 ± 10.96	76.45 ± 11.07
Height, cm	155.98 ± 4.45	157.32 ± 5.91	157.76 ± 5.94	157.49 ± 6.29
Waist circumference, cm	91.88 ± 9.91	90.75 ± 9.34	91.07 ± 8.66	92.16 ± 8.91
BMI (Kg/m^2^)	31.17 ± 4.37	30.55 ± 4.13	30.74 ± 3.87	30.86 ± 4.47
Body fat, %	38.85 ± 6.16	39.39 ± 5.43	39.45 ± 6.11	40.22 ± 6.29
Glucose, mg/dL	104.39 ± 10.3	96.66 ± 13.57	95.59 ± 7.58	97.83 ± 8.44
Total cholesterol, mg/dL	186.74 ±37.56	172.75 ± 34.36	171.00 ± 26.13	183.75 ± 24.84
HDL, mg/dL	41.74 ± 10.55	44.40 ± 10.58	41.26 ± 9.66	44.54 ± 8.44
LDL, mg/dL	110.59 ± 35.12	96.37 ± 40.76	96.72 ± 26.54	109.79 ± 22.99
Triglycerides, mg/dL	172.13 ± 71.43	159.89 ± 82.26	165.10 ± 80.22	154.03 ± 80.1

^1^ Values are means ± Standard deviation.

^2^ There were no significant differences between treatment groups in ANOVA.

PMR = Partial meal replacement, INU = Inulin, CON = Control group.

### Effect of treatments on anthropometry and body composition

**Figure 2:**
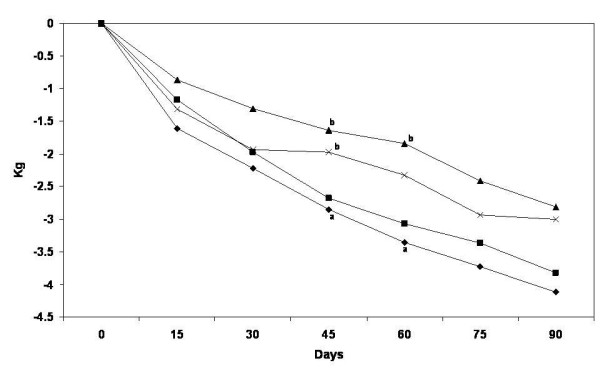
**Body weight change by treatment group.** Values are estimated mean changes adjusted for the initial value. ^a, b^ Different letters mean significant difference between treatments. PMR + I = Partial meal replacement with inulin, PMR = Partial meal replacement, INU = Inulin, CON = control.

**Figure 3:**
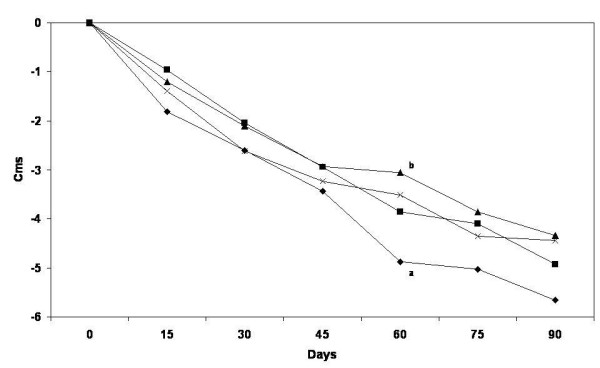
**Waist circumference change by treatment group.** Values are estimated mean changes adjusted for the initial value. Different letters mean significant difference between treatments. PMR = Partial meal replacement with inulin, PMR = Partial meal replacement, INU = Inulin, CON = control group.

All groups significantly reduced their weight, BMI, and waist and hip circumferences. Weight was significantly lower from baseline value in all treatment groups since the first post-treatment evaluation after 2wk, and weight loss was maintained along the trial. After 90 days, weight reduction of women in the PMR + I, PMR, INU and CON groups was [Mean (95%CI)]: –3.88 (−4.92, -2.84), -4.09 (−4.97, -3.20), -2.81 (−3.68, -1.93) and −2.89 (−3.85, -1.94) Kg, respectively. There were no significant differences in unadjusted changes between groups. The differences of adjusted changes found were as follows: After 45 days a greater reduction of BMI was observed in PMR group (mean; 95% CI) (−1.16; -1.40, -0.91 kg/m^2^) compared with INU (−0.68; -0.94, -0.42) and CON (−0.80; -1.06, -0.54) groups. At 60 days, BMI decreased more in PMR + I (−1.24; -1.56, -0.93) and PMR (−1.37; -1.66, -1.08) than in INU (−0.75; -1.05, -0.44). After 75 and 90 days no differences were observed between groups. Similarly, weight loss was greater at 45 days in PMR group (−2.85; -3.45, -2.25Kg) compared with INU (−1.64; -2.26, -1.01) and CON (−1.97; -2.59, -1.35) groups. And at 60 days, weight decreased more in PMR + I (−3.07; -3.84, -2.29) and PMR (−3.36; -4.07, -2.66) than in INU (−1.84; -2.59, -1.10) (Figure 2). Adjusted waist circumference reduction after 60 days was 1.8 cm greater, in PMR group (−4.87; -5.77, -3.96) compared with INU group (−3.06; -4.02, -2.11) (Figure 3). Body fat after the intervention was statistically similar than baseline.

### Effect of treatments on lipids and glucose concentration

Groups PMR + I, PMR, INU, significantly reduced by 21.5%, 19.3% and 20% respectively, their triglycerides concentration after 3 months of intervention (Table 3). No other significant changes were found in lipids or glucose concentrations.

**Table 3:** **Treatment effect on glucose and lipid profile**^***1***^

**Biochemical variables**	**PMR + I**	**PMR**	**INU**	**CON**
N	23	28	30	29
**Glucose, g/dL**				
Baseline	104.4 ± 10.3	96.7 ± 13.6	95.6 ± 7.6	97.8 ± 8.4
Final	101.7 ± 13.7	93.2 ± 13.3	96.0 ± 8.7	95.8 ± 7.9
Adjusted change^2,3^	−0.4 ± 9.4	−4.1 ± 9.1	−0.6 ± 9.1	−2.2 ± 9.0
**Total cholesterol, g/dL**				
Baseline	186.7 ± 37.6	172.8 ± 34.4	171.0 ± 26.1	183.8 ± 24.8
Final	176.6 ± 37.2	174.5 ± 30.0	176.3 ± 26.7	184.6 ± 26.9
Adjusted Change ^2^	−6.6 ± 24.1	−0.4 ± 24.0	2.4 ± 24.0	3.1 ± 24.0
**HDL, g/dL**				
Baseline	41.7 ± 10.5	44.4 ± 10.6	41.3 ± 9.7	44.5 ± 8.4
Final	38.8 ± 9.1	40.6 ± 9.7	41.7 ± 8.7	40.8 ± 10.1
Adjusted Change ^2^	−3.3 ± 6.6	−3.4 ± 6.6	−0.1 ± 6.6	−3.3 ± 6.6
**LDL, g/dL**				
Baseline	110.6 ± 35.1	96.4 ± 40.8	96.7 ± 26.5	109.8 ± 23.0
Final	110.7 ± 30.3	108.9 ± 25.8	108.3 ± 25.6	117.5 ± 24.0
Adjusted Change ^2^	4.4 ± 22.4	8.9 ± 22.4	8.1 ± 22.4	11.5 ± 22.4
**Triglycerides, g/dL**				
Baseline	172.1 ± 71.4	159.9 ± 82.3	165.1 ± 80.2	154.0 ± 80.1
Final	135.5 ± 49.6^3^	124.9 ± 55.0^3^	131.6 ± 57.2^3^	133.4 ± 47.2
Adjusted Change ^2^	−30.8 ± 42.2^3^	−36.4 ± 42.2^3^	−31.8 ± 42.2^3^	−25.6 ± 42.2

^1^ Values are means ± standard deviation.

^2^ Estimated mean adjusted for baseline value.

^3^ Change is different within treatment in pared T-Test, p < 0.05

No significant differences were found between treatments.

PMR = Partial meal replacement, INU = Inulin, CON = Control group.

### Food and nutrient intake

The energy, carbohydrates, protein, fat and cholesterol intake was significantly reduced in all groups (Table 4). Fat intake reduction was greater in PMR and PMR + I groups than in INU and CON groups, and fiber intake increased more in PMR + I and INU groups than in PMR and CON groups. Cholesterol intake was lower in PMR and PMR + I groups than CON group. Protein intake was higher in PMR + I and INU groups than the CON group.

**Table 4:** **Macronutrients intake at baseline and during the study**^***1***^

	**PMR + I**	**PMR**	**INU**	**CON**
N	21	26	27	28
**Energy, kJ**				
Baseline	8,431.5 ± 2,631.0	7,865.9 ± 3,060.4	7,327.5 ± 2,233.1	7,574.6 ± 2,041.8
Final	4,624.0 ± 1,454.9	4,688.4 ± 1,487.1	5,198.8 ± 1,986.1	4,607.7 ± 1,221.6
Adjusted change^2^	−3,807.5 ± 2,468.7	−3,177.5 ± 3,180.0	−2,128.8 ± 2,987.5	−2,966.9 ± 2,248.4
**Carbohydrates, g**				
Baseline	242.1 ± 75.6	247.4 ± 99.6	241.9 ± 75.4	216.0 ± 82.9
Final	144.9 ± 62.7^3^	143.9 ± 52.9^3^	138.9 ± 34.8^3^	161.6 ± 75.4^3^
Adjusted change^2^	−92.3 ± 57.3	−94.1 ± 57.4	−98.3 ± 57.3	−71.5 ± 57.7
**Protein, g**				
Baseline	80.2 ± 32.6	69.6 ± 28.3	65.4 ± 24.2	71.5 ± 16.9
Final	64.0 ± 14.2^3^	59.0 ± 11.3	64.9 ± 17.8	58.1 ± 19.5^3^
Adjusted change^2^	−7.9 ± 16.4	−12.1 ± 16.2	−5.9 ± 16.3	−13.1 ± 16.2
**Total fat, g**				
Baseline	85.0 ± 39.8	72.6 ± 36.2	67.7 ± 24.8	67.8 ± 25.6
Final	27.9 ± 12.1^3^	31.4 ± 25.5^3^	34.1 ± 19.1^3^	42.3 ± 19.5^3^
Adjusted change^2^	−45.6 ± 20.2 ^a^	−41.1 ± 19.9 ^a^	−38.0 ± 19.9	−29.8 ± 19.9 ^b^
**Cholesterol, mg**				
Baseline	264.4 ± 157.7	227.2 ± 116.4	200.3 ± 110.3	238.8 ± 101.5
Final	124.8 ± 112.4^3^	92.1 ± 38.3^3^	130.1 ± 66.1^3^	198.6 ± 172.3
Adjusted change^2^	−112.0 ± 110.3 ^a^	−138.2 ± 109.4 ^a^	−95.4 ± 110.3 ^b^	−33.7 ± 109.4 ^b^
**Total fiber, g**				
Baseline	13.9 ± 5.1	14.9 ± 9.5	13.6 ± 10.4	14.6 ± 7.1
Final	17.5 ± 6.1^3^	9.1 ± 4.0^3^	20.83 ± 6.3^3^	13.4 ± 5.1
Adjusted change^2^	3.5 ± 7.3 ^a^	−4.9 ± 7.4^b^	6.97 ± 7.5^a^	−0.75 ± 7.5 ^b^

^1^ Values are means ± Standard error.

^2^ Estimated mean adjusted for baseline value.

^3^ Change is different within treatment in pared T-Test, p < 0.05.

^a, b^ Different letters represent significant differences between treatments p < 0.05.

PMR = Partial meal replacement, INU = Inulin, CON = Control group.

Macronutrient and micronutrient intakes as percent of adequacy from the recommended intake by treatment group at baseline and during the intervention are shown in Table 5. Following the intervention PMR and PMR + I groups had a higher percent of adequacy of calcium, iron, zinc, vitamins C, B1, B2, B6, B12 niacin and folic acid than the CON group. Intakes of sodium, potassium, vitamin A and B12 were similar among treatment groups.

**Table 5:** **Percent of adequacy of recommended intake of nutrients in each treatment group at baseline and with intervention **^***1***^

**Nutrient**		**PMR + I**	**PMR**	**INU**	**CON**
Carbohydrates, %	Baseline	84.2 ± 11.8	88.9 ± 12.1	90.4 ± 9.3	85.4 ± 12.5
	Intervention	91.6 ± 7.8 2	90.4 ± 16.5	87.2 ± 9.9	90.0 ± 12.3
Protein, %	Baseline	106.4 ± 21.3 ^a^	100.2 ± 17.3	98.0 ± 12.5	113.9 ± 24.8 ^b^
	Intervention	163.9 ± 37.8 ^2,^^a^	151.1 ± 33.3 ^2, a^	158.2 ± 29.4 ^2^^a^	128.4 ± 23.6 ^2 b^
Fat, %	Baseline	95.4 ± 13.8	93.5 ± 14.6	92.1 ± 16.5	89.5 ± 15.6
	Intervention	74.3 ± 14.7 ^2^	73.2 ± 27.6 ^2^	80.5 ± 15.3 ^2^	82.4 ± 14.2
Calcium, %	Baseline	98.8 ± 26.9	92.1 ± 40.4	92.7 ± 40.3	92.7 ± 29.5
	Intervention	119.0 ± 20.3 ^2,a^	118.9 ± 37.7 ^2 a^	107.2 ± 35.8 ^a^	83.3 ± 31.3 ^b^
Iron, %	Baseline	68.4 ± 21.7	62.0 ± 34.8	60.3 ± 27.5	54.8 ± 17.9
	Intervention	71.8 ± 14.1 ^a^	69.8 ± 14.6 ^a^	40.7 ± 14.9 2 ^b^	47.1 ± 16.4 ^b^
Magnessium, %	Baseline	74.9 ± 37.9	80.6 ± 42.1	75.2 ± 44.2	81.1 ± 38.7
	Intervention	107.4 ± 26.7 ^2^^a^	107.2 ± 32.0 ^2 a^	62.2 ± 22.3 ^b^	68.7 ± 30.0 ^b^
Sodium, %	Baseline	99.6 ± 42.0	86.0 ± 48.2	90.0 ± 44.0	88.8 ± 43.7
	Intervention	68.7 ± 48.6 ^2^	63.9 ± 29.4 ^2 a^	69.8 ± 31.9 ^2^	85.3 ± 42.6 ^b^
Potassium, %	Baseline	47.1 ± 14.8	50.0 ± 21.3	48.4 ± 21.9	52.5 ± 10.2
	Intervention	49.0 ± 13.2	46.9 ± 12.1	54.2 ± 16.3	48.1 ± 14.1
Zinc, %	Baseline	47.3 ± 22.1	68.6 ± 96.8	48.4 ± 21.1	54.3 ± 21.1
	Intervention	94.0 ± 25.1 ^2 a^	92.6 ± 18.8 ^a^	43.7 ± 19.8 ^b^	38.5 ± 11.7 ^2 b^
Vitamin A, %	Baseline	228.7 ± 270.5	144.5 ± 91.5	175.9 ± 311.2	138.0 ± 89.0
	Intervention	195.5 ± 83.9	198.9 ± 80.1 ^2^	160.0 ± 73.4	272.3 ± 490.2
Vitamin C, %	Baseline	126.5 ± 72.3	143.8 ± 144.6	102.8 ± 64.6	140.0 ± 108.7
	Intervention	214.7 ± 78.5 ^2^^a^	197.7 ± 65.3 ^a,b^	113.6 ± 61.3 ^c^	158.7 ± 125.3 ^b^
Vitamin B1, %	Baseline	129.3 ± 53.3	123.7 ± 58.7	109.4 ± 44.3	104.3 ± 26.5
	Intervention	158.6 ± 27.5 ^a^	154.7 ± 37.0 ^2 a^	88.5 ± 31.3 ^b^	98.7 ± 38.3 ^b^
Vitamin B2, %	Baseline	166.8 ± 72.0 ^a^	136.7 ± 63.3	117.8 ± 40.5 ^b^	135.9 ± 29.8 ^b^
	Intervention	195.7 ± 35.7 ^a^	188.5 ± 39.6 ^2 a^	134.9 ± 49.1 ^b^	138.3 ± 81.5 ^b^
Niacin, %	Baseline	125.0 ± 54.0	115.9 ± 47.5	104.9 ± 46.6	109.6 ± 39.4
	Intervention	157.9 ± 37.7 ^a^	164.9 ± 38.7 ^2 a^	113.5 ± 33.9 ^b^	102.1 ± 43.1 ^b^
Vitamin B6, %	Baseline	98.7 ± 47.7	104.3 ± 55.1	86.3 ± 33.8	101.8 ± 35.0
	Intervention	163.5 ± 32.8 ^2^^a^	159.1 ± 34.6 ^2 a^	96.2 ± 34.7 ^b^	95.8 ± 39.6 ^b^
Folic Acid, %	Baseline	37.7 ± 13.8	38.3 ± 31.5	34.9 ± 23.0	32.5 ± 15.5
	Intervention	112.0 ± 28.5 2 ^a^	109.2 ± 27.4 ^2 a^	35.9 ± 17.7 ^b^	40.4 ± 17.9 ^b^
Vitamin B12, %	Baseline	290.2 ± 445.1 ^a^	206.0 ± 281.8	120.3 ± 139.9 ^b^	170.4 ± 274.5
	Intervention	228.5 ± 98.1	195.3 ± 52.1	153.5 ± 175.1	258.3 ± 723.1

^1^ Values are means ± Standard deviation.

^2^ Change from baseline to intervention is significant within treatment in pared T-Test, p < 0.05.

^a, b^ Different letters represent significant differences between treatments p < 0.05.

PMR = Partial meal replacement, INU = Inulin, CON = Control group.

## Discussion

Results of PMR effect on body weight have been controversial. Recent revisions and meta-analysis suggest that their use have a moderate effect resulting in weight loss of around 9%–10% of total body weight in the short term (3–6 months), and 6%–8% in the long term (> 1 years), when used as part of an overall low-energy diet plan [11,12,17,34]. Our results showed that the inclusion of PMR alone or in combination with inulin to a LCD was equally effective to reduce weight compared to LCD alone. After 90 days, both groups with PMR lost an average of 4 Kg or about 5.4% of initial weight. This reduction was about 28% higher than the reduction with the LCD alone but the difference was not statistically significant. Similarly to our results Noakes [35] compared a group of obese men with PMR and LCD with a group with LCD alone and found after 3 months, a loss of body weight of 6.6 Kg (6.9%) and 6.0 Kg (6.3%), respectively. Another study in obese women found after one year of treatment a weight reduction of 5.0 Kg (6.2%) with PMR plus LCD and 6.1 Kg (8%) with LCD alone; the difference was not statistically significant [10]. Two previous studies [11] found reductions in average weight of 7.1 kilograms after 3 months or 7.7 kilograms after one year with the inclusion of PMR to a LCD ; but in these studies the effect was significantly higher than LCD mainly because the effect of LCD was very low (1.3 and 3.4 kilograms, respectively). One recent study found a significant positive effect on weight reduction by the inclusion of PMR to a LCD [36]. Obese subjects lost in average 13.5 kilograms (12.3%) with LCD plus PMR compared with 6.5 kilograms (6.7%) with LCD alone after 16 weeks. In this study, subjects had an average BMI at the beginning of the study of 38 Kg/cm^2^, which was much higher than the baseline average BMI in our study of 31 Kg/cm^2^. This suggests that the inclusion of PMR to an LCD is more effective when subjects are “more obese”.

Treatments with PMR, PMR + I and INU significantly reduced triglycerides after 3 months of treatment. Both groups with inulin and PMR alone had an effect, so this could be attributed to inulin or its combination with a loss of body fat. Of 11 studies reviewed by Delzenne [13] on the effect of inulin on blood lipids, 4 studies did not find an effect of inulin on total cholesterol and triglycerides, 3 studies showed significant reduction in triglycerides, and 5 studies showed a modest reduction in total cholesterol and LDL cholesterol. Brighenti [21] conducted a meta-analysis that included studies and concluded that the intake of inulin was associated with significant decreases in serum triglycerides of 0.17 mmol/L (15 mg/dL) or 7.5%. Brighenti [16] found a marked reduction on plasma triglycerides and moderate decrease in plasma cholesterol in twelve healthy men consuming 9 g inulin/d. Letexier et al. [18] observed a 16% decrease in plasma triglycerides in eight subjects consuming 10 g inulin/d. In the present study, a significant reduction of 21.3%, 21.9% and 20.3% of plasma triglycerides was found with PMR + I, PMR and INU groups. In average, our subjects did not show elevated concentration of cholesterol or triglycerides at the beginning of the study; thus the effect that we found in reducing triglycerides could be more important in obese subjects that have high lipids concentrations as has been reported in other studies [19,20,37][38].

Total energy and carbohydrates intakes decreased similarly in all groups and fat intake, including cholesterol, was reduced significantly more in both groups with PMR compared with INU and CON groups. Ditschuneit [11] found similar results; PMR reduced cholesterol intake 17% more than a group with LCD alone and fat intake decreased significantly 48% in the PMR group and the control group decreased 19%. Also, Ashley [10] found a decrease in the intake of saturated fat and cholesterol by the inclusion of PMR. As expected, PMR + I and INU groups significantly increased total fiber intake from 13.9 to 17.5, and 13.6 to 20.8 g/d per day, respectively. An increase in dietary fiber intake is highly recommended in obese subjects [39]. These results suggest that PMR added with inulin can contribute with a reduction in fat intake and an increase in dietary fiber intake which could be beneficial in obese individuals with a higher risk of developing hyperlipidemia, or insulin resistance.

An important finding of this study is that PMR could contribute to increase the intake of essential nutrients, which is especially important during caloric restriction and in populations that need to reduce calories but that are at risk of having micronutrient deficiencies. In Mexico for example, there is a high prevalence of obesity, about 30% of adults and 16% of children less than 12 years are obese[4]; and in many individuals, obesity occurs simultaneously with some micronutrient deficiencies [6]; prevalence of some micronutrient deficiencies documented in Mexican adults are: 27% in zinc, 34% in vitamin E, 34% in vitamin C, 20% in vitamin B2 and 20% have iron deficiency [40-42]. Treatments with PMR increased intake of vitamins and minerals compared with LCD alone, especially calcium, iron, magnesium, zinc, vitamin B1, B2, B6, B12, niacin, folic acid and vitamin C and contribute to meet recommendations of such nutrients during LCDÂ´s . Because PMR is prepared with skimmed milk, the groups that included PMR increased milk and calcium intake. Calcium intake increased 20 and 30% in the PMR + I and PMR groups, respectively. Long term use of LCDÂ´s have been associated with bone resorption in obese adults due to low intake of calcium which can lead to bone demineralization [43]. Treatments with PMR increased intake of vitamins and minerals compared with LCD alone, especially calcium, iron, magnesium, zinc, vitamin B1, B2, B6, B12, niacin, folic acid and vitamin C and contribute to meet recommendations of such nutrients during LCDÂ´s . Because PMR is prepared with skimmed milk, the groups that included PMR increased milk and calcium intake. Calcium intake increased 20 and 30% in the PMR + I and PMR groups, respectively. Long term use of LCDÂ´s have been associated with bone resorption in obese adults due to low intake of calcium which can lead to bone demineralization [43]. This is one of the reasons why intake of calcium supplements and/or milk has been found to have beneficial effects on mineralization during prolonged LCDÂ´s [44,45]. Our study suggests that the use of PMRÂ´s could contribute to meet recommended intakes of nutrients specially when there is need to reduce calorie intake and in individuals with micronutrient deficiencies.

As it is common in dietary intake data, there was a considerable variability in the nutrient intake data, even when some statistical differences were found, some micronutrient comparisons were low powdered because the sample size was calculated upon body weight. In addition, the study did not find body fat change statistically different between treatments, it has been documented that other methods such as DEXA could have provided more accurate results [46]. Since this study was not intended to confirm the improvement of nutrient status, more studies are needed to evaluate if increased nutrient intake also increases micronutrient absorption and improves micronutrient status.

In conclusion, we found no additional effect of including PMR or a PMR added with inulin to a calorie restricted diet on changes in body weight, BMI or fat loss, than a LCD alone; however, intake of PMR, PMR added with inulin or inulin alone contribute to reduce plasma triglycerides during calorie restriction. In addition, treatment of obesity with LCD that includes a PMR significantly increased intake of essential amino acids, vitamins and minerals and contributed to meet daily recommendations. Results of the present study are particular relevant for populations that have increased prevalence of obesity and that at the same time have high prevalence of micronutrient deficiencies. This study points out the importance of using adequately fortified meal replacement products to ensure nutrient adequacy during energy intake restriction for weight loss.

## Abbreviations

PMR: Partial meal replacement; PMR + I: Partial meal replacement with inulin; INU: Inulin; CON: Control group; LCD: Low calorie diet; ANOVA: Analysis of Variance.

## Competing interests

This study was partially financed by Nucitec SA de CV. MAD was an employee at this company and did not receive any compensation for this study. JLR owns shares of this company. Both participated in the concept and design of the study and providing intellectual interpretation of the results.

## Authors’ contributions

ART conducted the fieldwork and wrote the initial draft of the paper; MCC contributed with the data analysis and its interpretation as well as with the writing of the manuscript; SG participated in the design of the study and quality assurance; MAD participated in the concept and design of the project; JLR participated in the concept and design and revised critically and intellectually the manuscript. All authors read and approved the final manuscript.
